# Long-Term Services and Supports in Supplemental Benefits in Medicare Advantage Plans

**DOI:** 10.1001/jamanetworkopen.2025.26406

**Published:** 2025-08-11

**Authors:** Deepon Bhaumik, David C. Grabowski

**Affiliations:** 1 Department of Health Care Policy, Harvard Medical School, Boston, Massachusetts

## Abstract

**Importance:**

Medicare Advantage (MA) plans have had the ability to offer long-term services and supports (LTSS) supplemental benefits (eg, in-home support services, adult day health services, caregiver support, home-based palliative care, and nonopioid pain management) since 2019. However, it is largely unknown how available these benefits currently are among MA plans.

**Objective:**

To examine the prevalence of LTSS supplemental benefits among MA plans and enrollment within these plans.

**Design, Setting, and Participants:**

This longitudinal cohort study used publicly available Centers for Medicare & Medicaid Services data on MA plan benefits and enrollment from January 2019 to April 2025 to examine changes in the offering of and access to LTSS supplemental benefits among MA plans as well as the enrollment within such plans. The analysis was limited to those MA plans that were identified as health maintenance organization or preferred provider organization plans. Data analyses were performed from November 2024 to March 2025.21.4

**Main Outcomes and Measures:**

MA plans offering LTSS supplemental benefits, the share of MA beneficiaries enrolled in a MA plan with LTSS benefits at the county level, and benefit generosity between newer and older MA plans.

**Results:**

In this study of 4521 MA plans in 2019 and 6614 MA plans in 2025, the number of MA plans offering any LTSS benefits increased from 581 to 814 from 2019 to 2025; however, the share of such plans slightly decreased from 12.9% to 12.3%. A mean (SD) of 21.4% (20.9%) of MA beneficiaries at the county level were enrolled in plans offering supplemental benefits in 2019, which decreased to 7.9% (12.7%) in 2025. The share of plans offering in-home support services, adult day services, home-based palliative care, and nonopioid pain management increased by 4.6, 0.02, 1.1, and 1.9 percentage points, respectively, whereas caregiver support services decreased by 5.6 percentage points. Lastly, when comparing newer and older MA plans offering LTSS benefits, newer MA plans offered 0.59 (95% CI, 0.48-0.70) more benefits than older MA plans.

**Conclusions and Relevance:**

In this cohort study, the availability of LTSS benefits within MA plans in 2025 was essentially no different than it was when benefits were first offered. The share of MA beneficiaries enrolled in a plan offering LTSS decreased during the same time frame, suggesting that there is still untapped potential for MA plans to offer LTSS supplemental benefits.

## Key Points

**Question:**

To what extent have Medicare Advantage (MA) plans provided long-term services and supports (LTSS) supplemental benefits over time?

**Findings:**

In this cohort study of 4521 MA plans in 2019 and 6614 MA plans in 2025, the availability of LTSS benefits within MA plans in 2025 was 12.3%, essentially the same as it was when benefits were first introduced in 2019 (12.9%). However, the share of MA beneficiaries enrolled in a plan offering LTSS decreased by 14 percentage points over the same time frame.

**Meaning:**

These results suggest that there is still a large untapped potential for LTSS to be offered through MA.

## Teaser

This cohort study examines the prevalence of and enrollment within Medicare Advantage plans with long-term services and supports (LTSS) supplemental benefits as well as plan generosity.

## Introduction

In 2018, the Centers for Medicare & Medicaid Services (CMS) announced the expansion of its definition of primary health-related supplemental benefits that could be covered by Medicare Advantage (MA) plans.^1^ Traditionally, supplemental benefits encompassed medical services, such as dental services, vision, and hearing, but as of 2019, MA plans have been able to offer nonmedical benefits, such as in-home support services, adult day health services, caregiver support (eg, respite care), home-based palliative care, and nonopioid pain management (eg, therapeutic massages).

Supplemental benefits have long been a major component of MA and annually amount to more than $60 billion in health care expenditures.^2^ This finding is particularly notable given that MA has recently become the prevailing source of Medicare coverage, with more than half of all Medicare beneficiaries enrolled in an MA plan in 2024.^3^ Importantly, these supplemental benefits are not available through traditional Medicare^4^ because supplemental benefits are financed from the savings, or rebates, generated by MA plans.^2^

Several of these new supplemental benefit offerings are considered long-term services and supports (LTSS). These services can be delivered in home, community, or institutional settings, such as nursing homes. Historically, LTSS has been outside the scope of services covered by Medicare, with beneficiaries typically relying on Medicaid, out-of-pocket payments, and unpaid caregiving from family members to meet their LTSS needs. More than half of adults older than 65 years require some form of LTSS, with a desire to age in place in the community as opposed to nursing homes.^2,5^ Increased provision of LTSS improves outcomes for patients and reduces health care costs,^6,7^ and providing LTSS in home or community settings can delay or potentially avoid the more expensive alternative of older adults being shifted to nursing homes.

Previous studies^7,8,9,10^ have found a limited initial uptake of these new supplemental LTSS benefits in MA plans, especially within the first year of implementation. In addition, the adoption of LTSS supplemental benefits is associated with a modest improvement in MA plan ratings.^11^ However, few studies^12,13,14^ have explored the increase (or decrease) of LTSS supplemental benefits among MA plans over time. Studying the differences between the initial and long-term impact of this policy is critical for understanding the scope of MA to provide LTSS to its beneficiaries. Similarly, understanding the differences between newer and older plans provides insight into learning and competition among MA plans offering supplemental LTSS benefits.^15,16,17^ In this study, we examine the evolution of MA plans offering LTSS supplemental benefits from 2019 to 2025. We focus on the prevalence of and enrollment within MA plans with LTSS supplemental benefits as well as plan generosity.

## Methods

### Data and Measures

For this study, we used publicly available Medicare Advantage/Part D Contract and Enrollment Data from the CMS from January 2019 to April 2025. We specifically used the Plan Benefit Package, county plan–level enrollment, and crosswalk datasets. The Plan Benefit Package dataset provided information on MA plan features, such as plan type and types of supplemental benefits covered. The county plan–level enrollment dataset specified the number of individuals enrolled in a specific MA plan per county. Because the CMS does not publish enrollment data with 10 or fewer observations due to Health Insurance and Portability and Accountability Act privacy laws, we recoded these county plan observations as having an enrollment of zero. At the plan level, 935 MA plans (20.7%) in 2019 and 1223 MA plans (18.5%) in 2025 had zero enrollment. The crosswalk datasets allowed us to link MA plans across years and observe the renewal status of plans as well as any plan consolidation or termination. This study followed the Strengthening the Reporting of Observational Studies in Epidemiology (STROBE) reporting guideline for reporting cohort studies. This cohort study was deemed not to be human participants research by the Harvard Medical School institutional review board; therefore, no informed consent was required.

We focused on the following LTSS supplemental benefits as consistent with previous studies: in-home support services, adult day health services, caregiver support, home-based palliative care, and nonopioid pain management.^9,10^ Other benefits, such as stand-alone memory fitness benefits, could not be identified in the data during the study period.

Every year when MA plans are reviewed, they are classified into 3 broad groups by the CMS as observed in the crosswalk dataset: new, renewal, or terminated. New MA plans typically refer to a plan that was added to an existing MA contract, but there are also plans that are created through a new MA contract. Within renewals, there are 4 categories for MA plans. They can be simply renewed, renewed and consolidated into another plan, renewed and expanded in a service area, or renewed and decreased in a service area.

We limited our analysis to those MA plans that were identified as health maintenance organization or preferred provider organization plans.^18^ We included special needs plans but excluded plans identified as Medicare-Medicaid and Program for All-inclusive Care for the Elderly because these plans are likely to be influenced by state-level factors.^10^ Analyses were performed from November 2024 to March 2025, using Stata, version 18.5 (StataCorp).

### Statistical Analysis

We first identified MA plans offering LTSS benefits (ie, in-home support services, adult day health services, caregiver support, home-based palliative care, or nonopioid pain management) and then categorized by type of benefit. We also examined access to LTSS benefits by calculating the share of MA beneficiaries enrolled in an MA plan with LTSS benefits at the county level. To do so, we divided the number of individuals enrolled in an MA plan offering LTSS benefits by the number of individuals enrolled in all MA plans for every county. We then measured the size (in terms of enrollment) of MA plans offering LTSS supplemental benefits. We next studied the composition of MA plans in terms of renewal status to understand the drivers behind annual changes in the number of MA plans offering LTSS supplemental benefits. For every year of our study, we identified MA plans that were terminated in the following year. We also compared plan generosity between newer and older MA plans. We defined plan generosity as the number of LTSS supplemental benefits offered by a plan. Older plans refer to plans that had offered LTSS benefits since 2019. Newer plans refer to plans that were categorized as new plans or initial contracts in 2020 and offering LTSS benefits. We also constructed additional definitions of newer plans for the years 2021 to 2025. In sensitivity analyses, we constructed alternate definitions of older plans. We used regression analysis, adjusting for plan-level factors (plan type, Special Needs Plan status, and plan size) as well as year-level fixed effects. We limited this analysis to plans that did not have any changes in their renewal status during the study period. Finally, we examined the share of MA plans with specific plan features for their LTSS benefits. We specifically focused on the following features: whether a plan had copayments for LTSS benefits, maximum plan benefit coverage (ie, the maximum dollar amount that a plan will pay for a benefit), prior authorization, and referral requirements. Statistical significance was set at 2-sided *P* < .05.

## Results

This study included 4521 MA plans in 2019 and 6614 MA plans in 2025. During the first 7 years (2019-2025) after the new rule, the share of MA plans offering any LTSS supplemental benefits slightly decreased from 12.9% (581 plans) to 12.3% (814 plans) (eFigure 1 in Supplement 1). From 2019 to 2020, a total of 108 fewer MA plans offered LTSS supplemental benefits, and 9 plans were terminated (eTable 1 in Supplement 1). From 2024 to 2025, a total of 263 fewer plans offered LTSS supplemental benefits, and 135 MA plans exited. The number of total MA plans increased from 4521 to 6614 during the same period. Before the CMS expansion rule, 4 MA plans (0.1% of all MA plans) offered LTSS supplemental benefits (Table 1). Specifically, 2 plans offered in-home support services, and 2 plans offered home-based palliative care. After an initial 3.9–percentage point decrease in 2020, the share of MA plans increased annually and was the highest in 2023, when 18.9% of plans (1307 plans) offered supplemental benefits. During the study period, the share of plans offering in-home support services, adult day services, home-based palliative care, and nonopioid pain management increased by 4.6 percentage points (from 2.6% to 7.2%), 0.02 percentage points (from 0.04% to 0.06%), 1.1 percentage points (from 0.5% to 1.6%), and 1.9 percentage points (from 0.5% to 2.4%), respectively. The share of plans offering caregiver support services decreased by 5.6 percentage points (which was due to one major insurer removing caregiver benefits from its list of covered benefits^7^) but increased 1.3 percentage points from 2020 to 2025.

**Table 1.** Descriptive Statistics of MA Plans Offering Supplemental Long-Term Services and Supports Benefits From 2018 to 2025

Year	Plans, No. (%)					
	Share of MA Plans	In-home support services	Adult day health services	Caregiver support	Home-based palliative care	Nonopioid pain management
2018	4 (0.1)	2 (0.05)	0	0	2 (0.05)	0
2019	581 (12.9)	117 (2.6)	2 (0.04)	419 (9.3)	23 (0.5)	22 (0.5)
2020	473 (8.9)	219 (4.2)	81 (1.5)	125 (2.4)	55 (1.1)	211 (4.0)
2021	662 (11.3)	383 (6.5)	104 (1.8)	87 (1.5)	109 (1.9)	163 (2.8)
2022	953 (14.7)	681 (10.5)	45 (0.7)	147 (2.3)	122 (1.9)	173 (2.7)
2023	1307 (18.9)	1015 (14.7)	36 (0.5)	245 (3.5)	131 (1.9)	178 (2.6)
2024	1077 (15.8)	701 (10.3)	5 (0.07)	298 (4.4)	154 (2.3)	165 (2.4)
2025	814 (12.3)	478 (7.2)	4 (0.06)	242 (3.7)	104 (1.6)	161 (2.4)

Abbreviation: MA, Medicare Advantage.

**Figure 1.**
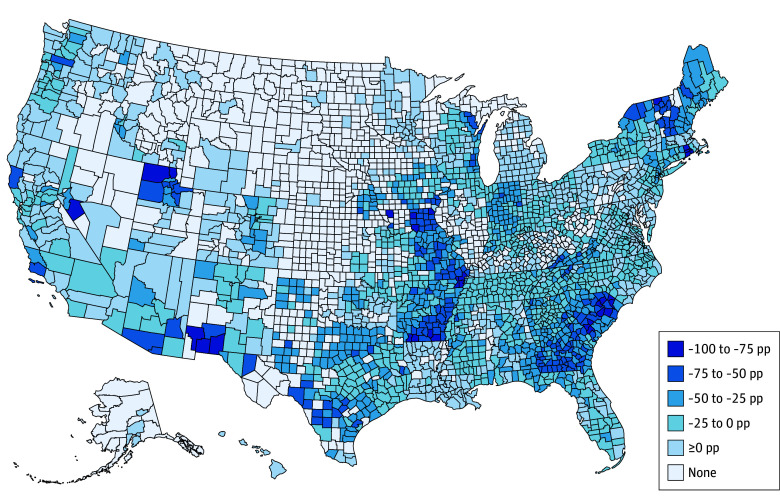
Change in the Share of Medicare Advantage Beneficiaries Enrolled in a Supplemental Long-Term Services and Supports Benefit Plan From 2019 to 2025 by US County pp indicates percentage points.

From 2019 to 2025, there was an overall decrease in the share of MA beneficiaries enrolled in plans offering LTSS supplemental benefits across US counties (Figure 1). There were noticeable decreases in counties in the Midwest, Northeast, and Southern regions of the US (eFigures 2 and 3 in Supplement 1). On average, counties had a 13.5–percentage point decrease in the share of their MA beneficiaries enrolled in plans offering LTSS benefits from 2019 to 2025 (eTable 2 in Supplement 1).

**Figure 2.**
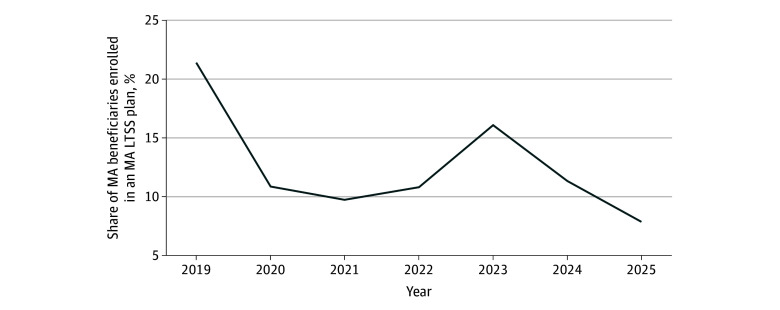
Share of Medicare Advantage (MA) Beneficiaries Enrolled in a Supplemental Long-Term Services and Supports (LTSS) Benefit Plan From 2019 to 2025 Across US Counties

From 2019 to 2021, the share of MA beneficiaries enrolled in an MA LTSS plan decreased from 21.4% to 9.8% across US counties (Figure 2). The enrollment share then increased to 16.1% in 2023 and decreased to 7.9% in 2025. Although the average MA LTSS plan size also decreased from 2019 to 2021 (eFigure 4 in Supplement 1), there was no noticeable change in the average plan size from 2023 to 2025.

When comparing newer and older MA plans offering LTSS supplemental benefits, MA plans that began operating in 2020 offered 0.53 (95% CI, 0.44-0.62) more LTSS benefits compared with MA plans offering LTSS benefits since 2019 (Table 2). Compared with MA plans offering LTSS benefits since 2019, MA plans that were created in 2021 offered 0.50 (95% CI, 0.43-0.57), those created in 2022 offered 0.64 (95% CI, 0.56-0.72), those created in 2022 offered 0.70 (95% CI, 0.62-0.78), those created in 2024 offered 0.85 (95% CI, 0.75-0.95), and those created in 2025 offered 0.97 (95% CI, 0.81-1.13) more LTSS benefits, respectively. Plans that began operating and offering LTSS benefits anytime from 2020 to 2025 offered 0.59 (95% CI, 0.48-0.70) more LTSS benefits than MA plans offering benefits since 2019. Newer plans, on average, offered anywhere from 4 to 7 times more LTSS supplemental benefits than older plans. Alternate constructions of the definition of older plans yielded similar results (eTable 3 in Supplement 1).

**Table 2.** Differences in Supplemental Long-Term Services and Supports Benefit Generosity Between Newer vs Older Medicare Advantage Plans

Plan	No. of supplemental benefits, mean (SD)		OLS estimate (95% CI)^c^	Plan-years, No.	
	Newer plans^a^	Older plans^b^		Newer plans	Older plans
2020 vs 2019	1.06 (0.76)	0.30 (0.64)	0.53 (0.44-0.62)^d^	385	2318
2021 vs 2019	0.94 (0.64)	0.26 (0.54)	0.50 (0.43-0.57)^d^	479	1818
2022 vs 2019	1.05 (0.56)	0.25 (0.52)	0.64 (0.56-0.72)^d^	463	1366
2023 vs 2019	1.07 (0.65)	0.22 (0.50)	0.70 (0.62-0.78)^d^	417	957
2024 vs 2019	1.15 (0.61)	0.17 (0.46)	0.85 (0.75-0.95)^d^	289	572
2025 vs 2019	1.27 (0.48)	0.19 (0.52)	0.97 (0.81-1.13)^d^	130	259
2020-2025 vs 2019	0.89 (0.72)	0.19 (0.52)	0.59 (0.48-0.70)^d^	546	259

Abbreviation: OLS, ordinary least squares.

^a^ Newer Medicare Advantage plans are defined as plans that have been categorized by the Centers for Medicare & Medicaid Services as new plans (ie, plans added to an existing contract) or initial contract (ie, new plans under a new contract) in the specified years.

^b^ Older Medicare Advantage plans are defined as plans offering supplemental benefits in 2019.

^c^ Regression models controlled for plan-level factors (eg, plan type, Special Needs Plan status, and plan size) and year-level fixed effects.

^d^ *P* < .001.

When examining specific plan features for LTSS supplemental benefits, the share of MA plans requiring copayments for LTSS benefits decreased by 18.7 percentage points from 2020 to 2025 (eTable 4 in Supplement 1). On the other hand, the share of MA plans requiring prior authorization or referrals for LTSS benefits increased from 2024 to 2025 by 5.5 and 1.2 percentage points, respectively. The share of MA plans with maximum benefit coverage for LTSS remained unchanged.

## Discussion

The increase of supplemental LTSS benefits within MA plans has oscillated over time, and the availability of these benefits within MA plans in 2025 is essentially no different than it was when benefits were first offered in 2019. Although the number of MA plans offering LTSS benefits has increased, the share of these MA plans has not changed, and the prevalence of the different types of LTSS have consistently remained low. Access to LTSS benefits has also decreased, from a mean (SD) of 21.4% (20.9%) of MA beneficiaries enrolled in a MA plan offering LTSS in 2019 to 7.9% (12.7%) in 2025. Lastly, newer plans are more generous in their offering of these extra benefits than older plans. Taken together, these results suggest that with the increase in MA enrollment over time, access to LTSS within MA has potentially become more limited.

One important distinction to note for the decrease in MA plans offering LTSS supplemental benefits is the difference between MA plans deciding to not offer these benefits and MA plans exiting the market. For example, although the decrease in the number of plans from 2019 to 2020 can be attributed to the fact that most of the plans stopped offering LTSS benefits, the decrease from 2024 to 2025 implies that a significant number of plans offering LTSS benefits in 2024 ceased operation in 2025.^15,16^

In the initial years of MA plans offering supplemental LTSS benefits, the decrease in enrollment among MA LTSS plans appears to be in part due to the decrease in plan size. However, since 2023, the share of MA beneficiaries enrolled in plans offering LTSS has decreased, whereas there were no noticeable changes in the average plan size. This finding seems to imply that, at least in more recent years, the decrease in enrollment in such plans is driven by the reduction in plans offering LTSS rather than existing plans losing market share. Recent research has found that an MA plan adding supplemental benefits did not decrease the likelihood of disenrollment.^19^ Although the share of plans offering LTSS surpassed its initial levels, the same was not true for the enrollment share. This finding appears to be consistent with the finding of a previous study^18^ that the addition of supplemental benefits was not associated with a change in the number of new MA enrollees.

The overall decrease in the share of MA beneficiaries enrolled in MA LTSS plans could be attributed to several reasons.^2^ First, it is unclear whether offering these benefits are financially viable for MA plans and whether adding new beneficiaries is profitable.^10^ As it stands, there is no additional funding from the CMS to provide these supplemental benefits.^20^ Second, it is unclear to what extent members value these LTSS supplemental benefits.^19^ The availability of these services could also be less appealing for dual-eligible patients, who were already eligible for and may already be receiving LTSS through Medicaid.^12,21^ Additional research on care coordination for this population could help understand these potential confusions.

Future research is also needed to understand the factors driving the reduction in the offering of LTSS benefits among MA plans since 2023, when LTSS offerings had previously mostly increased. One explanation could be the reductions in MA benchmarks that have occurred since 2023. These cuts may be causing insurers to reduce LTSS benefits.^2^ Some prior evidence suggests that a reduction in MA benchmarks modestly decreases plan generosity.^22^ Another potential explanation could be workforce shortages, given that the share of plans offering in-home support services has decreased greatly since 2023.^12^ On the other hand, workforce shortages may have no impact on plans offering such services because plans may incur limited costs from low use of these services. Beneficiary characteristics also potentially play a key role in determining the generosity of LTSS. A previous study^23^ found that MA plans exclusively serving dual-eligible populations offered more nonmedical benefits than general MA plans.

### Limitations

This study has several limitations. First, the classification of LTSS benefits within the plan benefit data was standardized from 2020 onward.^21^ Therefore, the number of LTSS benefits in 2019 may be an underestimate. Second, the CMS does not publish enrollment data with 10 or fewer observations due to privacy laws. Therefore, we recoded these county plan observations as having an enrollment of zero, which may lead to us underestimating MA enrollment. Third, although we could measure enrollment within MA plans, we could not measure use of LTSS within these plans. Fourth, we had limited access to plan-level characteristics and other plan features that may also contribute to the differences in LTSS generosity between older and newer plans. Fifth, the reporting of plan features from MA plans has been inconsistent over time, with only recent changes in reporting requirements from the CMS.^24^

## Conclusions

In this longitudinal cohort study, we found that the availability of LTSS benefits within MA plans in 2025 is essentially no different than it was when benefits were first offered in 2019. However, the share of MA beneficiaries enrolled in plans offering supplemental benefits has decreased during the same time frame, and newer plans offer more generous LTSS benefits than older plans, suggesting that there is still untapped potential for MA LTSS supplemental benefits.

## Notes

Supplement 1. **eTable 1.** Composition of Medicare (MA) plans offering supplemental long-term services and supports benefits by renewal status, from 2019 to 2025 **eTable 2.** Share of Medicare Advantage (MA) beneficiaries enrolled in a plan offering supplemental Long-Term Services and Supports (LTSS) benefits, 2019-2025, across U.S. counties **eTable 3.** Differences in Supplemental Long-Term Services and Supports Benefit Generosity between Newer vs Older Medicare Advantage (MA) Plans, alternate construction **eTable 4.** Plan Features for Long-Term Services and Supports benefits among Medicare Advantage plans, from 2020 to 2025 **eFigure 1.** Number of Medicare Advantage (MA) Plans offering Long-Term Services and Supports (LTSS) supplemental benefits, from 2019 to 2025 **eFigure 2.** Share of Medicare Advantage beneficiaries enrolled in a supplemental Long-Term Services and Supports benefit plan in 2019, by US county **eFigure 3.** Share of Medicare Advantage beneficiaries enrolled in a supplemental Long-Term Services and Supports benefit plan in 2025, by US county **eFigure 4.** Average Plan Size of Medicare Advantage (MA) Plans offering Long-Term Services and Supports (LTSS) supplemental benefits, from 2019 to 2025

Supplement 2.
Data Sharing Statement
