# Supplementary material for: Long-Term Services and Supports in Supplemental Benefits in Medicare Advantage Plans
Source: JAMA Netw Open. 2025 Aug 11;8(8):e2526406. doi: 10.1001/jamanetworkopen.2025.26406 (PMC12340655; doi:10.1001/jamanetworkopen.2025.26406)
Supplement: Supplement 1. — eTable 1. Composition of Medicare (MA) plans offering supplemental long-term services and supports benefits by renewal status, from 2019 to 2025 eTable 2. Share of Medicare Advantage (MA) beneficiaries enrolled in a plan offering supplemental Long-Term Services and Supports (LTSS) benefits, 2019-2025, across U.S. counties eTable 3. Differences in Supplemental Long-Term Services and Supports Benefit Generosity between Newer vs Older Medicare Advantage (MA) Plans, alternate construction eTable 4. Plan Features for Long-Term Services and Supports benefits among Medicare Advantage plans, from 2020 to 2025 eFigure 1. Number of Medicare Advantage (MA) Plans offering Long-Term Services and Supports (LTSS) supplemental benefits, from 2019 to 2025 eFigure 2. Share of Medicare Advantage beneficiaries enrolled in a supplemental Long-Term Services and Supports benefit plan in 2019, by US county eFigure 3. Share of Medicare Advantage beneficiaries enrolled in a supplemental Long-Term Services and Supports benefit plan in 2025, by US county eFigure 4. Average Plan Size of Medicare Advantage (MA) Plans offering Long-Term Services and Supports (LTSS) supplemental benefits, from 2019 to 2025 [file jamanetwopen-e2526406-s001.pdf]

# Supplemental Online Content

Bhaumik D, Grabowski DC. Long-term services and supports supplemental benefits in Medicare Advantage plans. *JAMA Netw Open*. 2025;8(8):e2526406. doi:10.1001/jamanetworkopen.2025.26406

**eTable 1**. Composition of Medicare (MA) plans offering supplemental long-term services and supports benefits by renewal status, from 2019 to 2025

**eTable 2**. Share of Medicare Advantage (MA) beneficiaries enrolled in a plan offering supplemental Long-Term Services and Supports (LTSS) benefits, 2019-2025, across U.S. counties

**eTable 3.** Differences in Supplemental Long-Term Services and Supports Benefit Generosity between Newer vs Older Medicare Advantage (MA) Plans, alternate construction

**eTable 4.** Plan Features for Long-Term Services and Supports benefits among Medicare Advantage plans, from 2020 to 2025

**eFigure 1.** Number of Medicare Advantage (MA) Plans offering Long-Term Services and Supports (LTSS) supplemental benefits, from 2019 to 2025

**eFigure 2.** Share of Medicare Advantage beneficiaries enrolled in a supplemental Long-Term Services and Supports benefit plan in 2019, by U.S. county

**eFigure 3.** Share of Medicare Advantage beneficiaries enrolled in a supplemental Long-Term Services and Supports benefit plan in 2025, by U.S. county

**eFigure 4.** Average Plan Size of Medicare Advantage (MA) Plans offering Long-Term Services and Supports (LTSS) supplemental benefits, from 2019 to 2025

This supplemental material has been provided by the authors to give readers additional information about their work.

© 2025 Bhaumik D and Grabowski DC. *JAMA Network Open.*

### eTable 1. Composition of Medicare (MA) plans offering supplemental Long-Term Services and Supports benefits by renewal status, from 2019 to 2025

| Year | Exit | Renewal Plan | Consolidated Renewal Plan | Other Renewal Type Plan | New Plan | Total |
|---|---|---|---|---|---|---|
| 2019 | 0 | 340 | 13 | 117 | 111 | 581 |
| 2020 | 9 | 268 | 12 | 97 | 96 | 473 |
| 2021 | 14 | 375 | 28 | 127 | 132 | 662 |
| 2022 | 26 | 595 | 47 | 159 | 152 | 953 |
| 2023 | 24 | 776 | 83 | 273 | 175 | 1307 |
| 2024 | 96 | 730 | 58 | 132 | 157 | 1077 |
| 2025 | 135 | 501 | 51 | 132 | 130 | 814 |

Notes: “Other Renewal Type Plans” refers to plans that were renewed and expanded its service area, or were renewed and decreased its service area

### eTable 2. Share of Medicare Advantage (MA) beneficiaries enrolled in a plan offering supplemental Long-Term Services and Supports (LTSS) benefits, 2019-2025, across U.S. counties

| Year | Share of MA beneficiaries |
|---|---|
| 2019 | 21.4% |
| 2020 | 10.9% |
| 2021 | 9.8% |
| 2022 | 10.8% |
| 2023 | 16.1% |
| 2024 | 11.3% |
| 2025 | 7.9% |

© 2025 Bhaumik D and Grabowski DC. *JAMA Network Open.*

**eTable 3. Differences in Supplemental Long-Term Services and Supports Benefit Generosity between Newer vs Older Medicare Advantage (MA) Plans, alternate construction**

| | Number of Supplemental Benefits | |
|---|---|---|
| | OLS Estimate[c] (95% Confidence Interval) | N (plan-years) |
| 2021 Plans[a] vs. 2019-2020 Plans[b] | 0.28*** (0.21, 0.34) | 3,383 |
| 2022 Plans vs. 2019-2020 Plans | 0.45*** (0.38, 0.52) | 2,639 |
| 2023 Plans vs. 2019-2020 Plans | 0.49*** (0.42, 0.57) | 1,941 |
| 2024 Plans vs. 2019-2020 Plans | 0.70*** (0.61, 0.80) | 1,209 |
| 2025 Plans vs. 2019-2020 Plans | 0.85*** (0.73, 0.97) | 547 |
| 2021-2025 Plans vs. 2019-2020 Plans | 0.53*** (0.43, 0.63) | 924 |

*p<0.1; **p<0.05; ***p<0.01

Notes: [a] “2021 Plans” (and the subsequent years) are “Newer MA Plans” which are defined as plans that have been categorized by CMS as “new plans” (i.e., plans added to an existing contract) or “initial contract” (i.e., new plans under a new contract) in the specified years.

[b] “2019-2020 Plans” are “Older MA Plans”, which are defined as plans offering supplemental benefits in 2019 or 2020.

[c] Regression models controlled for plan-level factors (plan type, Special Needs Plan status, plan size), as well as year-level fixed effects.

© 2025 Bhaumik D and Grabowski DC. *JAMA Network Open.*

**eTable 4. Plan Features for Long-Term Services and Supports benefits among Medicare Advantage plans, from 2020 to 2025**

| | 2020 | 2021 | 2022 | 2023 | 2024 | 2025 |
|---|---|---|---|---|---|---|
| Co-Payment, % | 59.4% | 44.9% | 48.3% | 38.2 | 41.1% | 40.7% |
| Maximum Plan Benefit Coverage, % | 4.4% | 5.7% | 1.3% | 2.1% | 4.1% | 4.7% |
| Prior Authorization, % | 0.0% | 0.0% | 0.0% | 0.0% | 18.2% | 23.7% |
| Referral Requirement, % | 0.0% | 0.0% | 0.0% | 0.0% | 5.9% | 7.1% |
| N (# of plans) | 473 | 662 | 953 | 1,307 | 1,077 | 814 |

Note: Percentages refer to the share of MA plans with the specified plan feature. The classification of LTSS benefits within the plan benefit data prior was only standardized from 2020 onwards, hence the exclusion of 2019 data.

© 2025 Bhaumik D and Grabowski DC. *JAMA Network Open.*

**eFigure 1. Number of Medicare Advantage (MA) Plans offering Long-Term Services and Supports (LTSS) supplemental benefits, from 2019 to 2025**

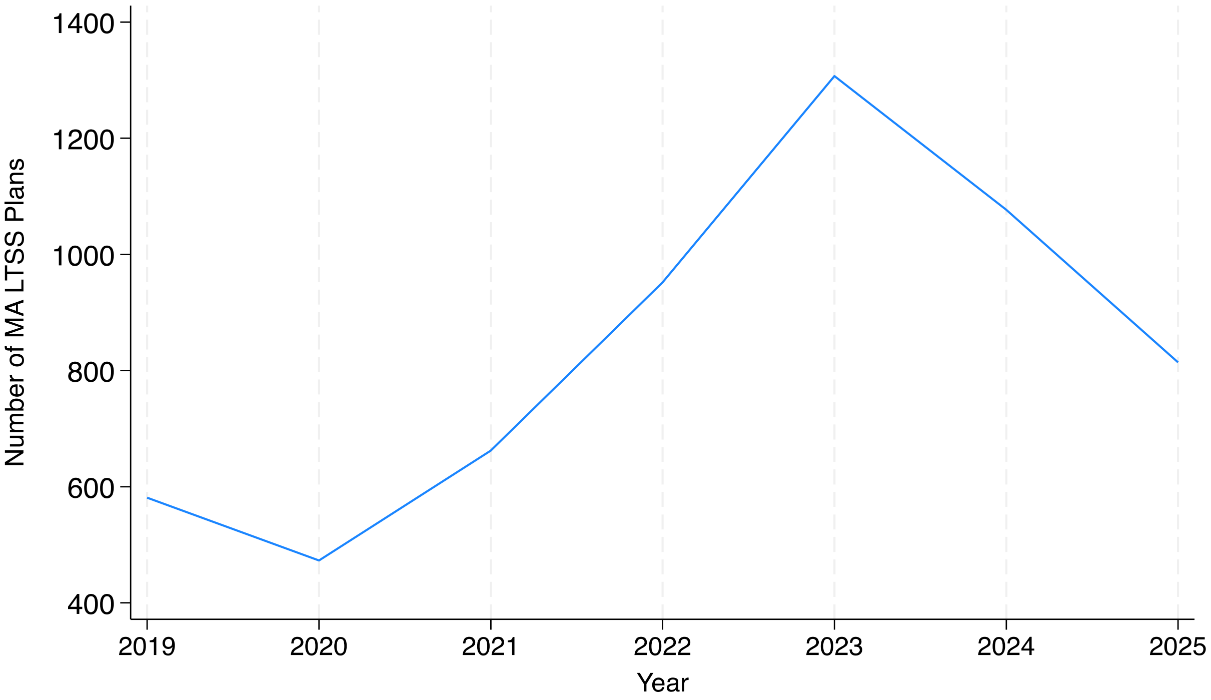


© 2025 Bhaumik D and Grabowski DC. *JAMA Network Open.*

**eFigure 2. Share of Medicare Advantage beneficiaries enrolled in a supplemental Long-Term Services and Supports benefit plan in 2019, by U.S. county**

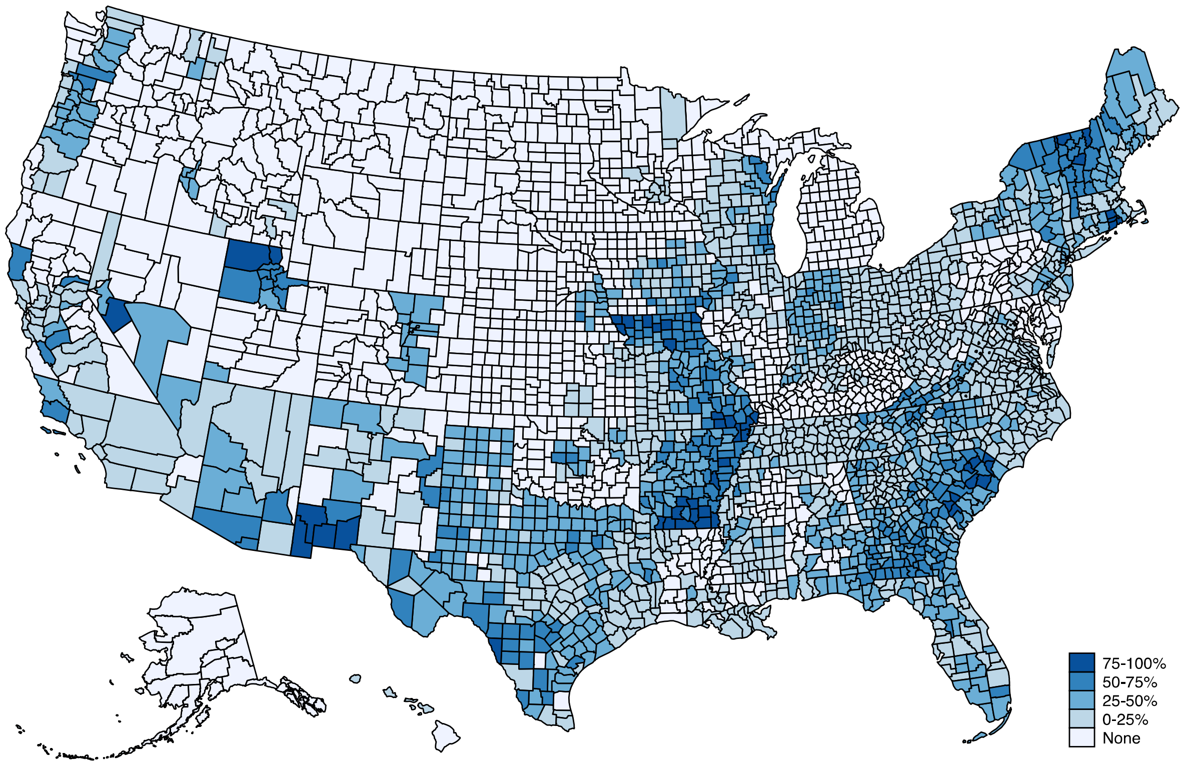


© 2025 Bhaumik D and Grabowski DC. *JAMA Network Open.*

**eFigure 3. Share of Medicare Advantage beneficiaries enrolled in a supplemental Long-Term Services and Supports benefit plan in 2025, by U.S. county**

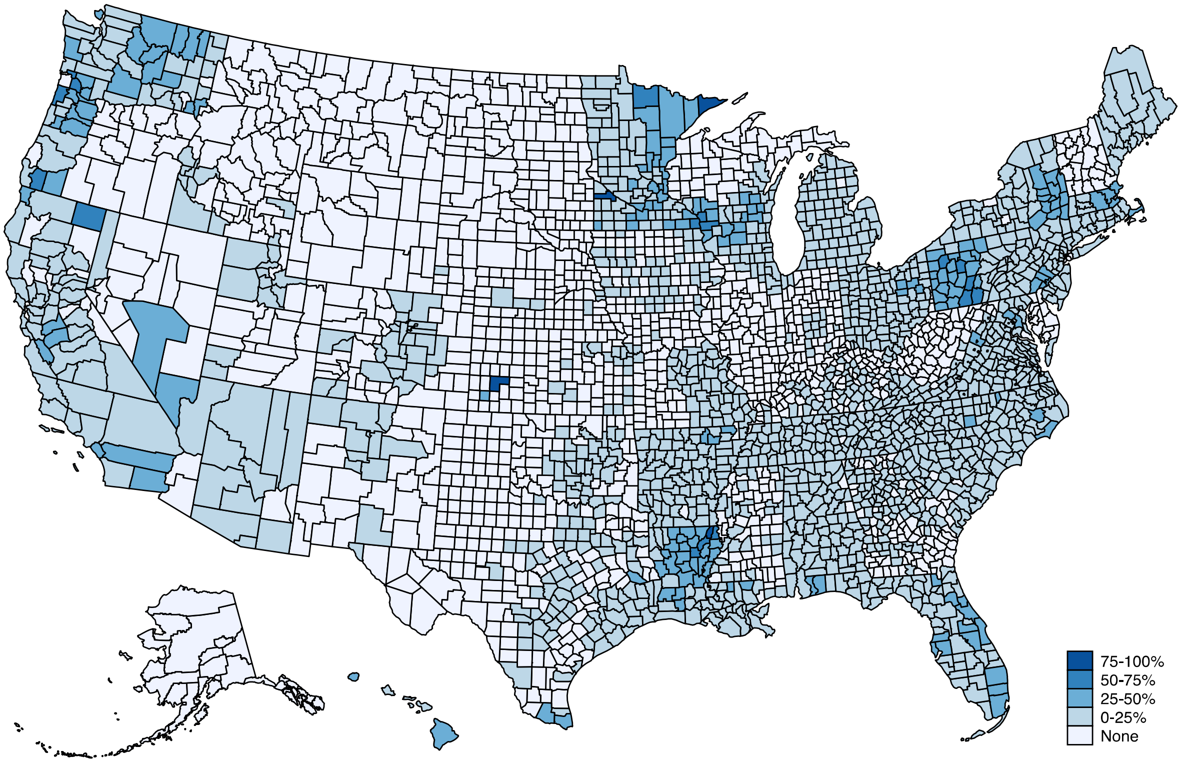


© 2025 Bhaumik D and Grabowski DC. *JAMA Network Open.*

**eFigure 4. Average Plan Size of Medicare Advantage (MA) Plans offering Long-Term Services and Supports (LTSS) supplemental benefits, from 2019 to 2025**

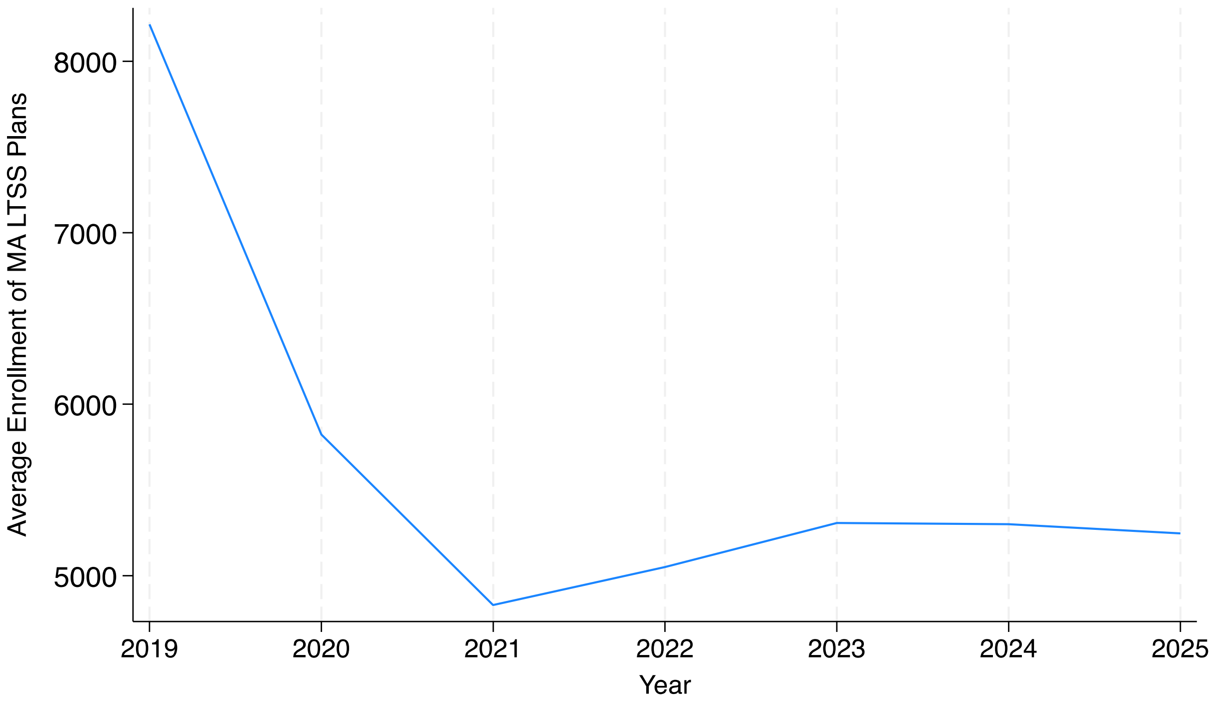


© 2025 Bhaumik D and Grabowski DC. *JAMA Network Open.*
