# Supplementary material for: Long-Term Services and Supports in Supplemental Benefits in Medicare Advantage Plans
Source: JAMA Netw Open. 2025 Aug 11;8(8):e2526406. doi: 10.1001/jamanetworkopen.2025.26406 (PMC12340655; doi:10.1001/jamanetworkopen.2025.26406)
Supplement: Supplement 2. — Data Sharing Statement [file jamanetwopen-e2526406-s002.pdf]

Data Sharing Statement

Bhaumik. Long-Term Services and Supports in Supplemental Benefits in Medicare Advantage Plans. *JAMA Netw Open*. Published August 11, 2025.
doi:10.1001/jamanetworkopen.2025.26406

Data
Data available: No

Additional Information
Explanation for why data not available: This study used publicly available Medicare Advantage/Part D Contract and Enrollment Data from CMS which is available online.
